# REALITIES OF DIVERSITY, INCLUSION, AND BELONGING IN ASSISTED LIVING: SAFETY, CONNECTION, AND COMMUNITY

**DOI:** 10.1093/geroni/igae098.3885

**Published:** 2024-12-31

**Authors:** Jacklyn Kohon, Dani Himes, Sarah Dys

**Affiliations:** Portland State University, Portland, Oregon, United States; Portland State University, Portland, Oregon, United States; Portland State University, Portland, Oregon, United States

## Abstract

This study centers the voices and experiences of those working and living in assisted living, residential care, and memory care (AL/RC) settings in Oregon to understand how workplace diversity, equity, inclusion, accessibility, and belonging (DEIAB) can promote well-being. From January to April 2024, we conducted individual and focus group interviews with a total of 68 people, including 25 direct care staff, 9 former direct care staff, 9 administrators, 7 management representatives and 18 current residents from 23 AL/RC communities. We highlight six primary themes: 1) Care staff face discrimination, physical and verbal abuse from residents and colleagues; 2) those experiencing discrimination or not belonging were more likely to be from a minoritized group; 3) there is inconsistency in the existence, awareness, and currentness of diversity, equity, and inclusion policies, making implementation challenging; 4) company culture can support feelings of connection and belonging among staff; 5) care staff rely on peers and coworkers for support; and 6) workplaces need infrastructure that reflects language diversity of residents and care staff. Participants noted discrimination can happen overtly and subtly. In many instances, overt discrimination can be easier to address and handle, while subtle forms of discrimination happen daily and many times are invisible or look like unspoken norms. Recommendations include comprehensive DEIAB policies with clear implementation plans, DEIAB training programs for residents, investing in linguistic accessibility resources, and training administrators and managers in inclusive leadership practices.

